# Extracellular matrix and cyclic stretch alter fetal cardiomyocyte proliferation and maturation in a rodent model of heart hypoplasia

**DOI:** 10.3389/fcvm.2022.993310

**Published:** 2022-11-28

**Authors:** Matthew C. Watson, Corin Williams, Raymond M. Wang, Luke R. Perreault, Kelly E. Sullivan, Whitney L. Stoppel, Lauren D. Black

**Affiliations:** ^1^Department of Biomedical Engineering, Tufts University, Medford, MA, United States; ^2^Department of Mechanical Engineering, Tufts University, Medford, MA, United States; ^3^Cellular, Molecular, and Developmental Biology Program, Sackler School for Graduate Biomedical Sciences, Tufts University School of Medicine, Boston, MA, United States

**Keywords:** cardiomyocytes, proliferation, heart hypoplasia, extracellular matrix (ECM), mechanical stretch

## Abstract

**Introduction:**

Birth defects, particularly those that affect development of the heart, are a leading cause of morbidity and mortality in infants and young children. Babies born with heart hypoplasia (heart hypoplasia) disorders often have a poor prognosis. It remains unclear whether cardiomyocytes from hypoplastic hearts retain the potential to recover growth, although this knowledge would be beneficial for developing therapies for heart hypoplasia disorders. The objective of this study was to determine the proliferation and maturation potential of cardiomyocytes from hypoplastic hearts and whether these behaviors are influenced by biochemical signaling from the extracellular matrix (ECM) and cyclic mechanical stretch.

**Method:**

Congenital diaphragmatic hernia (CDH)-associated heart hypoplasia was induced in rat fetuses by maternal exposure to nitrofen. Hearts were isolated from embryonic day 21 nitrofen-treated fetuses positive for CDH (CDH+) and from fetuses without nitrofen administration during gestation.

**Results and discussion:**

CDH+ hearts were smaller and had decreased myocardial proliferation, along with evidence of decreased maturity compared to healthy hearts. In culture, CDH+ cardiomyocytes remained immature and demonstrated increased proliferative capacity compared to their healthy counterparts. Culture on ECM derived from CDH+ hearts led to a significant reduction in proliferation for both CDH+ and healthy cardiomyocytes. Healthy cardiomyocytes were dosed with exogenous nitrofen to examine whether nitrofen may have an aberrant effect on the proliferative ability of cardiomyocyte, yet no significant change in proliferation was observed. When subjected to stretch, CDH+ cardiomyocytes underwent lengthening of sarcomeres while healthy cardiomyocyte sarcomeres were unaffected. Taken together, our results suggest that alterations to environmental cues such as ECM and stretch may be important factors in the pathological progression of heart hypoplasia.

## Introduction

Congenital diaphragmatic hernia (CDH) is a serious birth defect that occurs in ~1 in 2,500–3,000 live births. Only ~16% of prenatally diagnosed CDH patients are expected to survive past the first year of life (1). Failure of the diaphragm to close permits intrusion of visceral organs into the thoracic cavity and subsequent compression of the developing heart and lungs. Heart and/or lung hypoplasia is associated with a particularly poor prognosis in CDH (2). Interestingly, patients born with mild to moderate heart hypoplasia exhibit restored heart growth after surgical repair of CDH (3); however, the underlying mechanisms are unknown. A better understanding of the factors that influence heart growth during development is not only critical for improving therapies for children with heart defects but would also be invaluable to the field of cardiac regeneration as a whole.

Cardiomyocyte proliferation plays a key role in cardiac growth during fetal development (4) and may provide an explanation for restored heart growth in repaired CDH. In other forms of heart hypoplasia, such as Hypoplastic Left Heart Syndrome, there is evidence that cardiomyocyte proliferation is severely diminished (5), although this has not been established in CDH-associated heart hypoplasia. Cardiomyocyte behavior can be regulated by a variety of biochemical and biophysical cues, such as the extracellular matrix (ECM) (6, 7), growth factors (6, 8), cell-cell contacts (9), substrate stiffness (10, 11), and mechanical stretch (12). Although current data is limited, studies suggest that at least some of these signals may be altered in the developing hypoplastic heart (13).

It is intriguing to consider the possibility that cardiomyocyte proliferation can be recovered in heart hypoplasia if pathological conditions are removed, or healthy biochemical/biophysical environments are restored. The experimental manipulation of mechanical loading in embryonic zebrafish (14) and chick hearts (15) motivated the use of fetal aortic annuloplasty in severe HLHS in an attempt to restore normal blood flow and, subsequently, left heart growth (16). In this study, we hypothesized that cardiomyocyte proliferation is reduced in CDH-associated heart hypoplasia, but that the cells retain the ability to proliferate if they are removed from the hypoplastic environment. We used the nitrofen model of CDH in rats, which is thought to be similar in etiology and phenotype to human CDH (17–19). In addition to studying proliferation in native hearts, we isolated cardiac cells and assessed their response to two external cues that have been implicated in heart hypoplasia defects: the biochemical signaling of the ECM, which is thought to be structurally immature in heart hypoplasia (20, 21), and cyclic stretch, which is diminished in heart hypoplasia (12). Our *in vitro* culture systems allowed us to systematically study these cues independently, which would not be possible *in vivo*. We found that CDH+ cardiomyocytes were more proliferative than healthy cardiomyocytes when placed in culture, and that ECM and cyclic stretch (1 Hz, 5% amplitude) differentially regulated proliferation and maturation.

## Materials and methods

### Nitrofen model of congenital diaphragmatic hernia

All animal procedures were performed in accordance with the Institutional Animal Care and Use Committee at Tufts University and the NIH Guide for the Care and Use of Laboratory Animals. Pregnant Sprague-Dawley rats at gestational day E10 (purchased from Charles River Laboratories, Wilmington, MA) were subjected to short Isoflurane anesthesia and immediately gavaged with a single dose of 100 mg nitrofen (Wako Pure Chemical Industries, Japan) dissolved in 2 ml olive oil. Control animals received olive oil alone, similar to previously described methods (13). Pregnancy then progressed until E21, at which point the dams were sacrificed by CO_2_ inhalation. E21 was chosen as the time point as this was immediately pre-birth and it has been previously shown that the defects caused by CDH are postnatally lethal. The fetuses were harvested for the studies described below.

### Fetal heart harvest

Immediately after harvest, fetuses were placed on ice, and euthanized by decapitation. The chest wall was carefully opened above the diaphragm and the heart and lungs were removed. The diaphragm was then checked for the presence of CDH. Nitrofen treatment at E10 resulted in CDH in ~80–85% of the fetuses, similar to what others have found (22). As so few nitrofen-treated CDH negative hearts were found (~2–4 out of 12–16 hearts per litter), they were not included in the present study. CDH was never detected in control fetuses. Nitrofen-treated CDH positive (CDH+) and control (“healthy”) hearts were weighed prior to further characterization.

### Histology and imaging of native heart sections

Whole hearts were fixed by immersion in 4% paraformaldehyde at 4°C overnight. After washing with phosphate buffered saline (PBS), the samples were cryo-protected in sucrose solution (30% wt/vol), and then embedded in Tissue Tek optimum cutting temperature (OCT) compound. The hearts were sectioned (thickness of 7 μm) on a Leica CM 1950 CryoStat. Heart slices were stored at −20°C until use. OCT compound was removed by washing with PBS prior to staining with hematoxylin and eosin (H&E) to visualize gross heart structure. Hearts were imaged on a Keyence BZ-X700 fluorescent microscope using a color camera. The thickness of the compact zone of the left ventricular free wall was measured with ImageJ using H&E stained sections.

### ECM composition

A subset of CDH+ and healthy hearts were used to determine extracellular matrix (ECM) composition by liquid chromatography tandem mass spectrometry (LC-MS/MS), similar to our previous studies of cardiac ECM (7). Immediately after weighing, freshly isolated whole hearts were decellularized in 0.1% sodium dodecyl sulfate (SDS) for 48 h at room temperature with agitation on an orbital shaker. The resulting ECM was then incubated in 0.1% TritonX-100 (Amresco, Solon, OH) for ~3–4 h, washed with distilled water, frozen, and lyophilized overnight (Labconco, Kansas City, MO). The ECM of individual fetal hearts was solubilized in 200 μl urea solution (5 M urea, 2 M thiourea, 50 mM DTT, 0.1% SDS) (23) for 20 h with agitation. Finally, the ECM was precipitated in acetone and analyzed *via* LC-MS/MS within 24 h at the Beth Israel Deaconess Medical Center Mass Spectrometry Core Facility. Among the samples (*N* = 3 for each condition), 34 unique ECM components were identified and their relative percentages in the total ECM composition was determined from spectrum counts.

### Immunocytochemistry

To study proliferation and sarcomere development in the native healthy and CDH+ hearts, sections were stained for nuclei (Hoechst 33258), Ki67 (Abcam, rabbit polyclonal), and sarcomeric α-actinin (Sigma, mouse monoclonal). Briefly, the samples were washed with PBS, then blocked with 5% donkey serum and 1% bovine serum albumin (BSA) for 1 h at room temperature. Incubation with primary antibodies was for 1 h, followed by 3 PBS washes, then incubation with secondary antibodies (AlexaFluor 488 donkey anti-rabbit IgG, AlexaFluor 555 donkey anti-mouse IgG; Invitrogen) for 1 h. After washing with PBS, the samples were imaged on an Olympus IX8I microscope with Metamorph Basic software (version 7.7.4.0, Molecular Devices). Image analysis to determine cardiomyocyte proliferation and sarcomere length was carried out as described below in sections Cell proliferation measurements and Cardiac cell culture with exogenous nitrofen.

### Cardiac gene expression by quantitative PCR

A panel of cardiac genes was analyzed by PCR. RNA was isolated from fetal hearts using the RNeasy^®^ Mini Kit (Qiagen Sciences, Germantown, MD) per the manufacturer's instructions. RNA concentration was determined using a Nanodrop 2000 Spectrophotometer (Thermo Scientific, Waltham, MA) and purity was assessed by the ratio of the 260/280 absorbance readings. Samples with high purity were then used to make cDNA using the High Capacity cDNA Reverse Transcription Kit (Applied Biosystems, Foster City, CA) per the manufacturer's instructions. cDNA was loaded into the wells of a MicroAmp^®^ Optical 96-well reaction plate with 10 μl of 2 × TaqMan^®^ Gene Expression Master Mix and 1 μl of predesigned 20 × TaqMan^®^ Gene Expression Assay primers for the specific gene of interest (Applied Biosystems, Foster City, CA) diluted in nuclease-free water to a final volume of 20 μl. Samples were probed for gene expression related to contractile function (MYH6, MYH7, TNNT2, TNNI3, ACTN2, ATPA2), gap junction signaling (GJA1, CDH2) and early markers of the cardiac lineage (GATA4, GATA6, NKX2-5). Expression of each gene was normalized to GAPDH values. Quantitative RT-PCR was performed using the Mx3000P QPCR System (Agilent Technologies, Lexington, MA) with incubation parameters of 2 min at 50°C, 10 min at 95°C and 40 cycles of 15 s at 95°C followed by 1 min at 60°C. Ct values were determined by the software provided with the Mx3000P QPCR System and differences in mRNA expression were calculated by the 2^−Δ*Δct*^ method (24) based on validation tests performed by Applied Biosystems.

### Cardiac cell isolation and culture

Cells from CDH+ and healthy control hearts were isolated according to our previously described methods (25). Briefly, hearts were isolated from euthanized fetal pups at E21, the ventricles were minced, and the tissue underwent 7 × 7 min digestions in collagenase type II (Worthington Biochemical Corp, Lakewood, NJ) and sterile PBS supplemented with 20 mM glucose. Cells were counted with a hemocytometer and seeded at a density of 100,000 cells/cm^2^ into tissue culture polystyrene 48-well plates. The culture medium contained 15% fetal bovine serum (FBS) in Dulbecco's Modified Eagle Medium (DMEM) with 1% penicillin-streptomycin and was changed every 2 days. The cells were fixed and stained with Hoechst, Ki67, and α-actinin at various time points, imaged, and analyzed as described below in sections Cell proliferation measurements and Cardiac cell culture with exogenous nitrofen.

### Cell proliferation measurements

Cell numbers and proliferation were determined using custom pipelines in CellProfiler (release 11710, the Broad Institute). Total cell nuclei were determined from the Hoechst stain and proliferating cells (Ki67+ nuclei) were determined from the Ki67 stain. The α-actinin stain used to label cardiomyocytes was used as a “mask” to identify cardiomyocyte-specific nuclei and proliferation. Total cell and cardiomyocyte density was calculated using the total imaged area (converted to mm^2^) for each sample. Cardiomyocyte-specific proliferation was measured as the percentage of proliferating cells that were also positive for sarcomeric α-actinin [(Ki67+ α-actinin+)/Ki67+].

### Cardiac cell culture with exogenous nitrofen

To determine whether nitrofen would affect the behavior of healthy cardiomyocytes, cardiomyocytes freshly isolated from neonatal hearts were seeded onto 12 well plates at a density of 50,000 cells/cm^2^. Cells were cultured in serum containing medium until beating was observed. Once beating was observed, cells were treated with medium containing nitrofen at 50 and 100 ug/ml. Dosages were determined by estimating the concentrations of nitrofen delivered to each rat fetus. Serum containing medium was used as a control. For normalization purposes, a subset of each group was fixed at Cells were treated on days 1 and 2, and wells from each group were fixed on either 1 day prior to the first nitrofen treatment or 3 days after the beginning of nitrofen treatment. Cells were stained, imaged, and analyzed for proliferation as described above in section Cell proliferation measurements.

### Sarcomere measurements

Sarcomere length has been used as a measure of cardiomyocyte maturation (10, 26, 27). To adequately visualize sarcomeres, images of α-actinin staining at 40 × magnification were acquired. Analysis was performed using ImageJ software (NIH, version 1.45s). When organized sarcomeres were observed, a line was manually drawn across multiple sarcomeres perpendicular to alignment. The “Plot Profile” function was used to display the staining intensity across the line. The number of sarcomeres in a given length was counted and the average measured sarcomere length was calculated. Sarcomeres were also categorized according to the following definitions: “developing” (sarcomeres measuring < 1.8 μm in length); and “mature” (≥1.8 μm) (28).

### Cardiac cell culture on ECM

ECM from healthy and CDH+ hearts was obtained as described above and solubilized at a concentration of 10 mg/ml in a solution containing 1 mg/ml pepsin and 0.1 M HCl (7, 29). The ECM solution was neutralized with NaOH, immediately coated onto 48-well plates at a density of 50 μg/cm^2^ and allowed to dry in a sterile tissue culture cabinet overnight. Prior to cell seeding, the ECM was washed three times with sterile PBS. Cells freshly isolated from healthy and CDH+ hearts were then seeded onto the coated plates at a density of 100,000 cells/cm^2^. To avoid the potential confounding effects of serum on proliferation, cells were cultured in a serum-free medium that contained the following: 50/50 mixture of DMEM and Ham's F12 Nutrient Mix (Invitrogen), 0.2% (wt/vol) bovine serum albumin (BSA) (Sigma), 0.5% (vol/vol) insulin–transferrin–selenium-X (Invitrogen) and 1% penicillin–streptomycin (Invitrogen), with 10 mM L -ascorbic acid (Sigma) added fresh at every feeding (30). Cells were fed on days 1 and 3, and wells from each group were fixed on either day 1 or day 4 in culture. Cells were stained, imaged, and analyzed for proliferation as described above in section Cell proliferation measurements.

### Cardiac cell culture with cyclic mechanical stretch

To determine the effects of cyclic mechanical stretch on cardiomyocyte behavior, cells were cultured on a custom-built cell culture membrane stretching device (31). Fetal cardiac cells isolated at E21 from healthy and CDH+ hearts were seeded at 1 × 10^6^ cells per well in 6-well Bioflex^®^ culture plates. The experimental set-up was similar to previously described methods with slight modifications (31). The Bioflex^®^ culture plate membranes were pre-coated with collagen type I by the manufacturer. We found that an additional surface treatment of 4 μg/cm^2^ human plasma Fibronectin (Millipore, Billerica MA) in DMEM applied overnight at 37°C with mild orbital plate agitation was necessary to create a consistent surface coating for cell adhesion. Cells were initially cultured under static conditions for 4 days post-isolation to ensure strong adhesion to the membrane and then stretched for 3 days on the custom-built system. Membranes were deformed based on a standard left ventricular volumetric loading waveform with 1 Hz frequency and 5% amplitude. Control samples were not subjected to stretching (“static”). The underside of the Bioflex^®^ culture membranes was lubricated with a silicone lubricant (Loctite, Düsseldorf, Germany) to minimize friction with the plunger during stretch. Cells were fed culture medium containing 10% horse serum, 2% FBS, and 1% penicillin-streptomycin in DMEM. Medium was changed and lubricant was reapplied every 2 days. At the end of the experiment, samples were fixed, stained, and imaged as described above. Analysis included proliferation and sarcomere measurements as described above in sections Cell proliferation measurements and Cardiac cell culture with exogenous nitrofen. In addition, subset of wells was subjected to gene expression analysis as described in section Cardiac gene expression by quantitative PCR.

### Statistical analysis

Statistical analysis was performed using analysis of variance (ANOVA) and Tukey's *post-hoc* test or the unpaired Student's t-test, as appropriate. Differences were considered statistically significant for *p* < 0.05. Statistical testing was carried out using GraphPad Prism 9 (GraphPad Software, San Diego, CA).

## Results

### Altered gross morphology in CDH+ hearts

**Figure 1:**
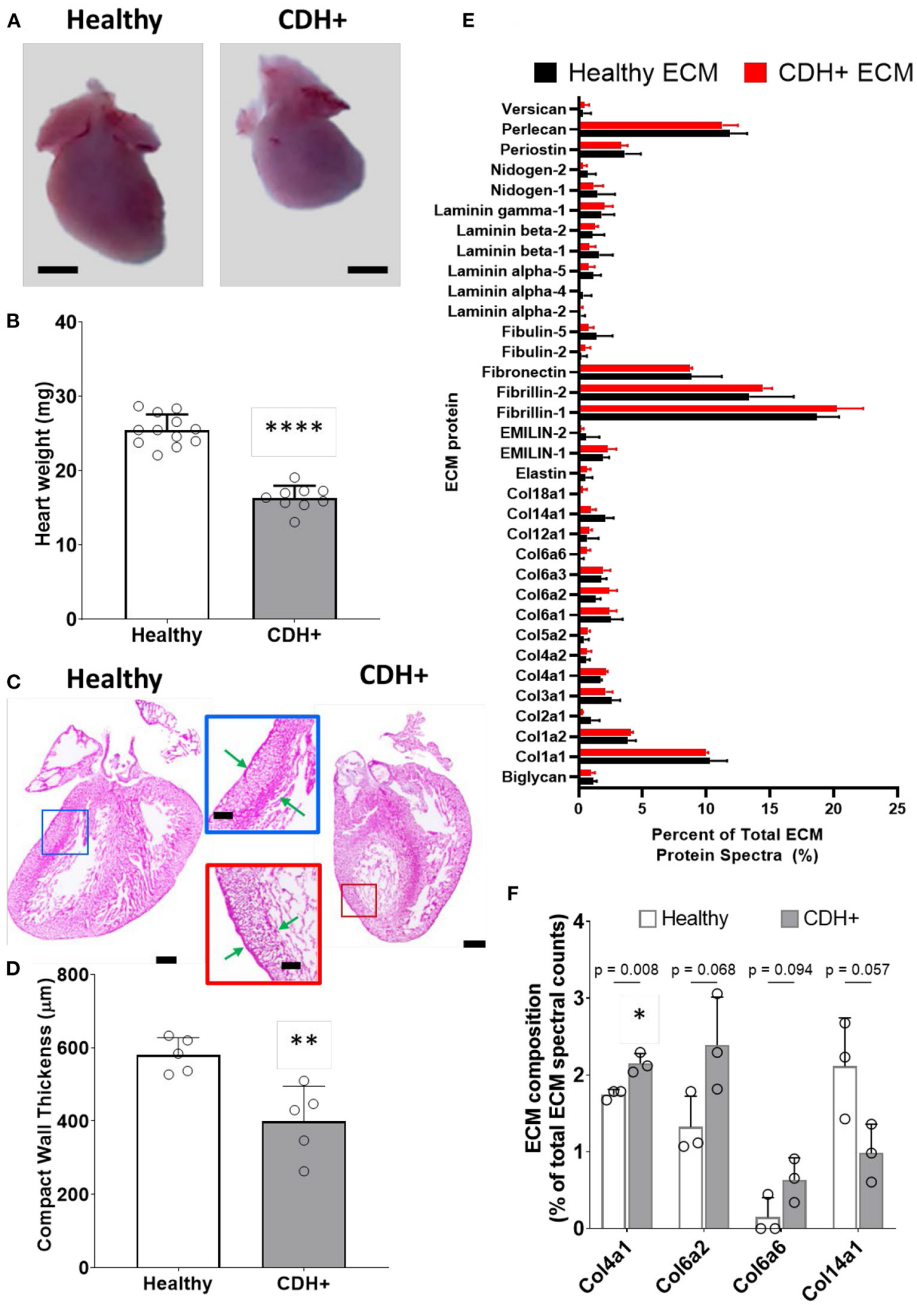
Altered heart morphology in the rat model of CDH. **(A)** Representative images of E21 hearts from healthy controls and nitrofen-treated CDH+ fetuses. Scale bar represents 1 mm. **(B)** Heart mass measurements (mean ± S.D.). **** Denotes *p* < 0.0001 from unpaired t-test. **(C)** Representative H&E stained sections of healthy and CDH+ hearts. Insets show measurements of LV compacted free wall thickness (green arrows). Scale bars = 500 μm for whole heart sections and 200 μm for insets. **(D)** Measurements of left ventricle (LV) free wall thickness (*n* = 5) (mean ± S.D.). ** Denotes *p* < 0.01 from unpaired t-test. **(E)** All proteins detected during LC-MS/MS proteomics analysis of healthy and CDH+ heart ECM. Data are presented as the percent of total ECM protein spectra (mean ± S.D.). **(F)** Data for proteins that are significantly different or close to it between healthy and CDH+ hearts. *p*-values for each comparison between healthy and CDH+ hearts using an unpaired t-test are shown above the grouped bars. Again, data is presented as percent of total ECM protein spectra.

Upon isolation at E21, we found that CDH+ hearts were smaller and had significantly decreased mass compared to healthy controls (Figures 1A,B). Qualitatively, H&E staining suggested that the CDH+ hearts were morphologically immature, with many still exhibiting the ventricular groove, thinner/less compacted ventricular walls, and more trabeculation compared to healthy hearts (Figure 1C). Measurements of the compact zone of the left ventricular free wall (Figure 1C, inset denoted by arrows) indicated that CDH+ hearts had thinner compact myocardium compared to healthy hearts (400 ± 96 vs. 580 ± 48 μm; Figure 1D*, p* < 0.01). ECM was also altered in CDH+ hearts as determined by LC-MS/MS (Figure 1E), particularly in some of the lower abundance components. Specifically, significant increases in the relative abundance of Collagen IV (*p* < 0.01) and nearly significant increases in Collagen VI peptides and decreased Collagen XIV (*p* < 0.1 in all cases) were found in CDH+ vs. healthy hearts (Figure 1F, Table 1). High abundance proteins such as Collagen I, Fibrillin-1, and Fibronectin were not significantly different.

**Table 1:** Relative ECM composition.

**ECM protein**	**Healthy**		**CDH**+		***p*-value**	**CDH**+ **fold change w.r.t. Healthy**	
	**Mean**	**±**	**Mean**	**±**		**Mean**	**±**
Biglycan	1.15	0.27	0.97	0.32	0.50	0.84	0.28
Col1a1	10.30	1.34	10.01	0.15	0.73	0.97	0.01
Col1a2	3.86	0.63	4.09	0.16	0.57	1.06	0.04
Col2a1	0.98	0.66	0.32	0.02	0.16	0.33	0.02
Col3a1	2.58	0.67	2.13	0.53	0.42	0.83	0.21
Col4a1	1.75	0.06	2.15	0.13	0.01	1.23	0.07
Col4a2	0.60	0.27	0.66	0.34	0.84	1.09	0.57
Col5a2	0.42	0.38	0.75	0.14	0.23	1.76	0.33
Col6a1	2.53	0.93	2.38	0.59	0.83	0.94	0.23
Col6a2	1.32	0.40	2.39	0.63	0.07	1.81	0.47
Col6a3	1.78	0.41	1.93	0.57	0.73	1.08	0.32
Col6a6	0.15	0.26	0.63	0.29	0.09	4.27	1.92
Col12a1	0.68	0.88	0.86	0.19	0.74	1.27	0.28
Col14a1	2.11	0.63	0.98	0.38	0.06	0.47	0.18
Col18a1	0.00	0.00	0.32	0.33	0.17	Inf	Inf
Elastin	0.54	0.54	0.64	0.29	0.80	1.18	0.54
EMILIN-1	1.91	0.49	2.29	0.67	0.47	1.20	0.35
EMILIN-2	0.60	1.03	0.21	0.18	0.56	0.35	0.31
Fibrillin-1	18.68	1.74	20.25	2.06	0.37	1.08	0.11
Fibrillin-2	13.38	3.47	14.45	0.71	0.63	1.08	0.05
Fibronectin	8.83	2.39	8.71	0.19	0.94	0.99	0.02
Fibulin-2	0.24	0.41	0.54	0.38	0.40	2.28	1.61
Fibulin-5	1.39	1.27	0.77	0.40	0.46	0.55	0.29
Laminin alpha-2	0.19	0.32	0.11	0.20	0.76	0.61	1.05
Laminin alpha-4	0.37	0.65	0.00	0.00	0.37	0.00	0.00
Laminin alpha-5	1.14	0.62	0.75	0.50	0.45	0.66	0.44
Laminin beta-1	1.62	1.07	0.85	0.47	0.32	0.53	0.29
Laminin beta-2	1.07	0.94	1.28	0.25	0.73	1.20	0.23
Laminin gamma-1	1.81	0.99	2.03	0.64	0.76	1.13	0.35
Nidogen-1	1.49	1.36	1.17	0.76	0.74	0.79	0.51
Nidogen-2	0.68	0.67	0.32	0.33	0.44	0.47	0.48
Periostin	3.63	1.25	3.32	0.52	0.71	0.91	0.14
Perlecan	11.86	1.35	11.26	1.22	0.60	0.95	0.10
Versican	0.36	0.62	0.45	0.39	0.84	1.25	1.08

Determined from spectral counts, data are percentages of total ECM spectra. Last column is fold change of CDH+ ECM components with respect to Healthy. Those in red show down regulation below 0.5-fold while those in green show upregulation greater than 1.5-fold.

### CDH+ hearts exhibit reduced cell proliferation and cardiomyocyte maturity

**Figure 2:**
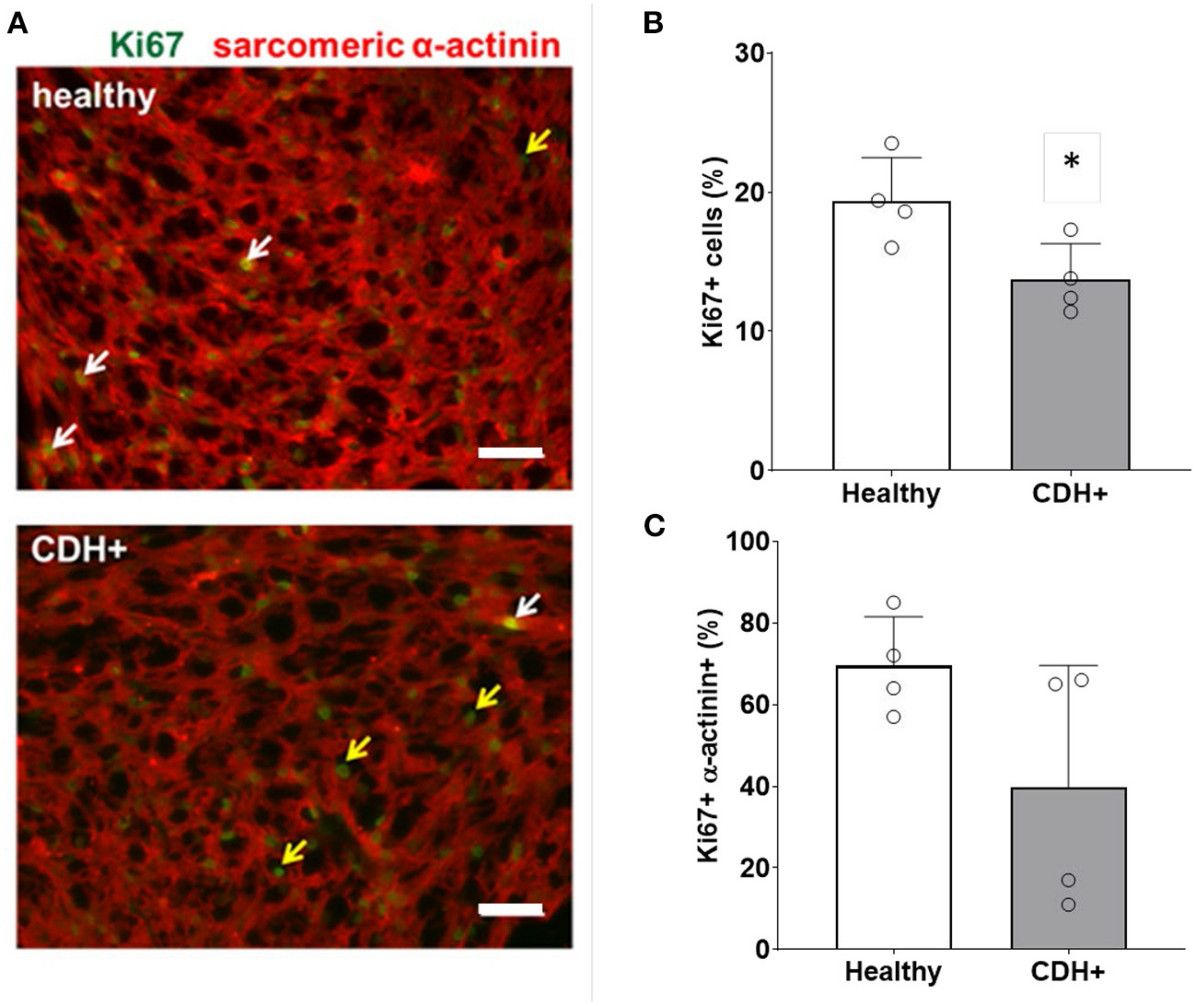
Proliferation measurements in healthy and CDH+ hearts. **(A)** E21 heart sections stained for Ki67 (green) and sarcomeric α-actinin (red); examples of Ki67+ cardiomyocytes (white arrows), Ki67+ non-myocytes (yellow arrows), scale bars = 50 μm. **(B)** Total cell proliferation shown as percent of cells expressing Ki67. * Denotes *p* < 0.05. **(C)** Cardiomyocyte-specific proliferation (the percentage of Ki67+ cells that were also α-actinin+).

**Figure 3:**
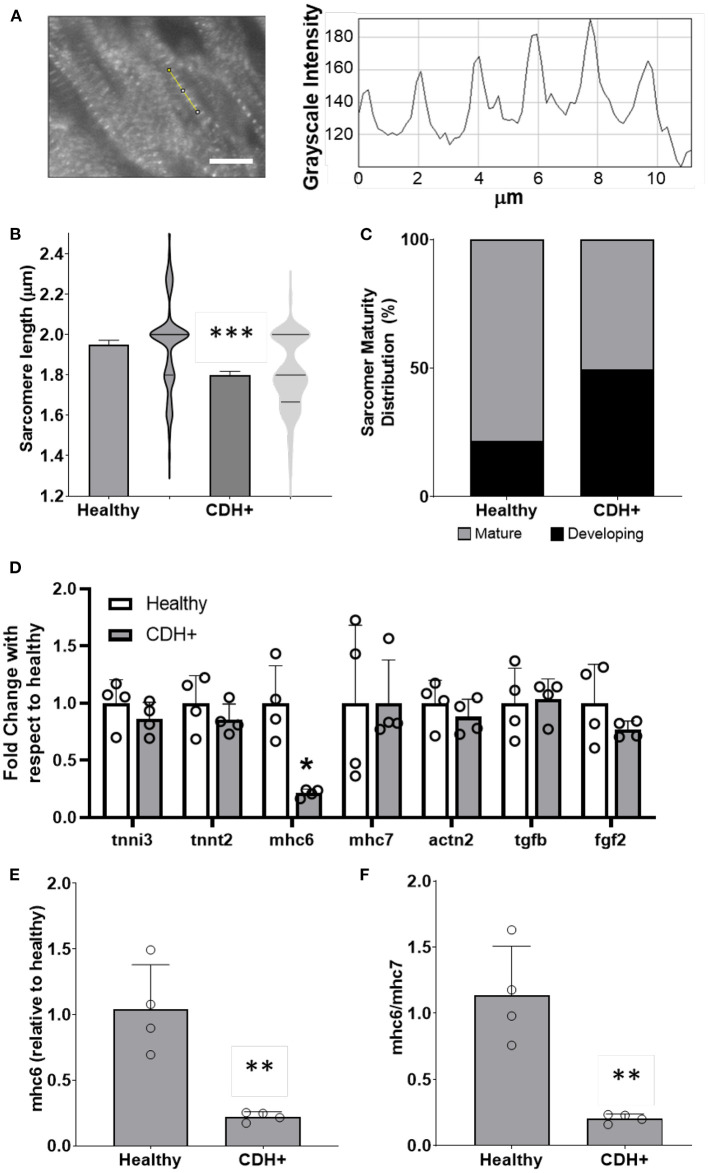
Maturation assessments in healthy and CDH+ hearts. **(A)** Example of sarcomere staining and measurement in native heart sections. A line is drawn perpendicular to the sarcomeres (yellow) and the profile is plotted in ImageJ. Peak-to-peak distances represent sarcomere lengths. Scale bar represents 10 μm. **(B)** Average sarcomere length measurements (mean ± S.E.M. on left, violin plots showing the distribution of the data on right, *n* > 80 individual cells per condition). *** Denotes *p* < 0.001. **(C)** A greater percentage of sarcomeres were mature (≥1.8 μm) in healthy vs. CDH+ hearts. **(D)** Gene expression data for panel of genes investigated. * *p* < 0.05 w.r.t. healthy. **(E)** Gene expression of αMHC (mhc6) as well as the ratio of α to β isoforms (mhc6/mhc7) and **(F)** was decreased in CDH+ hearts (*n* = 4). In both cases ** denotes *p* < 0.01.

To determine whether CDH+ hearts exhibited reduced proliferation, native heart sections were stained for Ki67 to identify proliferating cells and sarcomeric α-actinin to label cardiomyocytes (Figure 2A). Cell proliferation was significantly decreased in CDH+ hearts compared to healthy controls (Figure 2B). Particularly in CDH+ hearts, many non-myocytes appeared to be Ki67+ (Figure 3A*, yellow arrows*). Therefore we determined whether proliferation was decreased specifically in the cardiomyocyte population. Although cardiomyocyte proliferation (as determined by Ki67 + α-actinin + cells) showed a decreasing trend in CDH+ hearts, it was not statistically significant (*p* = 0.07) (Figure 2C).

To assess cardiomyocyte maturity, we analyzed sarcomere lengths and gene expression in native hearts. Average sarcomere length was significantly lower in CDH+ hearts compared to healthy (Figures 3A,B). In line with this data, a greater proportion of measured sarcomeres were “mature” (≥1.8 μm) in healthy vs. CDH+ hearts (~80 vs. ~50%, respectively; Figure 3C). Taken together, the sarcomere measurements imply that cardiomyocytes were less mature in CDH+ hearts compared to healthy. We also investigated a panel of cardiac genes (Figure 3D) and found that *mhc6*, the gene for myosin heavy chain α which is more abundant in the maturing or adult rat heart (32, 33), was significantly down-regulated in CDH+ hearts relative to healthy (Figure 3E). Furthermore, the ratio of mhc6 to mhc7, which has been used to assess cardiomyocyte maturity (34), was significantly decreased in CDH+ hearts (Figure 3F).

### CDH+ cardiomyocytes in culture remain immature and become proliferative

**Figure 4:**
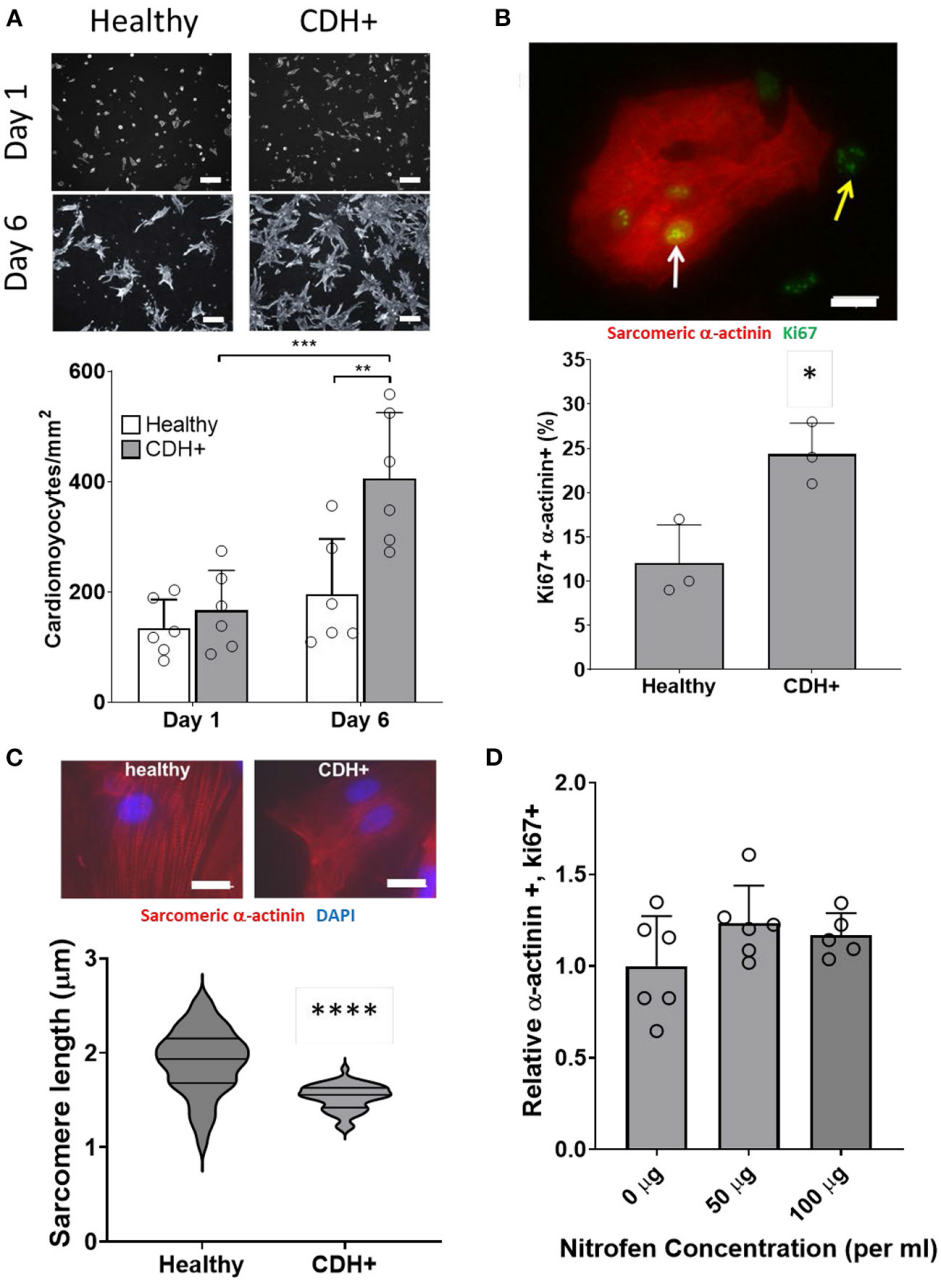
Characterization of CDH+ cardiomyocytes in culture. **(A)** Representative images of cardiomyocytes (α-actinin stain in gray scale) after 1 day or 6 days in culture (top). Cardiomyocyte density at 1 and 6 days (bottom). Graph shows mean ± SD (*n* = 6). ** Denotes *p* < 0.01 w.r.t. Healthy cells at Day 6. *** Denotes *p* < 0.001 w.r.t. CDH+ at 24 h. **(B)** Cardiomyocyte proliferation (Ki67+ α-actinin+) at 2 days. Inset shows example of Ki67+ cardiomyocyte (white arrow) and non-myocyte (yellow arrow). Graph shows mean ± SD (*n* = 3). * Denotes *p* < 0.05. **(C)** Representative images of sarcomere staining in healthy and CDH+ cardiomyocytes where Red is cardiac α-actinin and blue is the nuclear marker DAPI (top). Sarcomere length measurements (bottom). **** Denotes *p* < 0.001. **(D)** Proliferative cardiomyocytes at 72 h post-treatment with exogenous nitrofen. Data is normalized by relative expression of α-actinin+ and Ki67+ at 24 h pre-treatment. Graph shows mean ± SD (*n* = 5).

To determine whether proliferation remained low or could be recovered upon removal from the hypoplastic environment, we isolated cells from CDH+ and healthy hearts and cultured on standard tissue culture plastic. After 1 day cardiomyocyte density was similar for both conditions, but after 6 days there were significantly more cardiomyocytes in the CDH+ population compared to the healthy population (Figure 4A). Whereas the healthy cardiomyocytes did not significantly increase in number from 1 to 6 days (fold change of 1.45), the CDH+ cardiomyocytes increased 2.4-fold (*p* = 0.0025). At 2 days, Ki67 was detected in 24% of the CDH+ cardiomyocytes, compared to only 12% in the healthy, demonstrating that the CDH+ cells were indeed more proliferative (*p* < 0.02, Figure 4B). We also found that sarcomeres were significantly smaller in the CDH+ cardiomyocytes after 6 days, suggesting that these cells remained less mature than healthy cells (Figure 4C).

### Culture with exogenous nitrofen does not affect proliferation in healthy cardiomyocytes

In order to determine whether alterations in cardiomyocyte proliferation in the CDH+ animals were due to a direct effect of nitrofen, we treated cardiomyocytes with nitrofen and assessed proliferation over the course of 3 days. For these studies, freshly isolated cardiomyocytes were cultured with 0 mg, 50 mg, and 100 mg of nitrofen. After 72 h of culture, we observed no significant differences in in ki-67+ cardiomyocytes that were treated with exogenous nitrofen compared to cardiomyocytes cultured without nitrofen (Figure 4D), indicating that the responses of the cardiomyocytes from the CDH+ hearts were likely the result of alterations to the biochemical and biophysical properties of the ECM.

### CDH+ cardiac ECM inhibits cardiomyocyte proliferation

**Figure 5:**
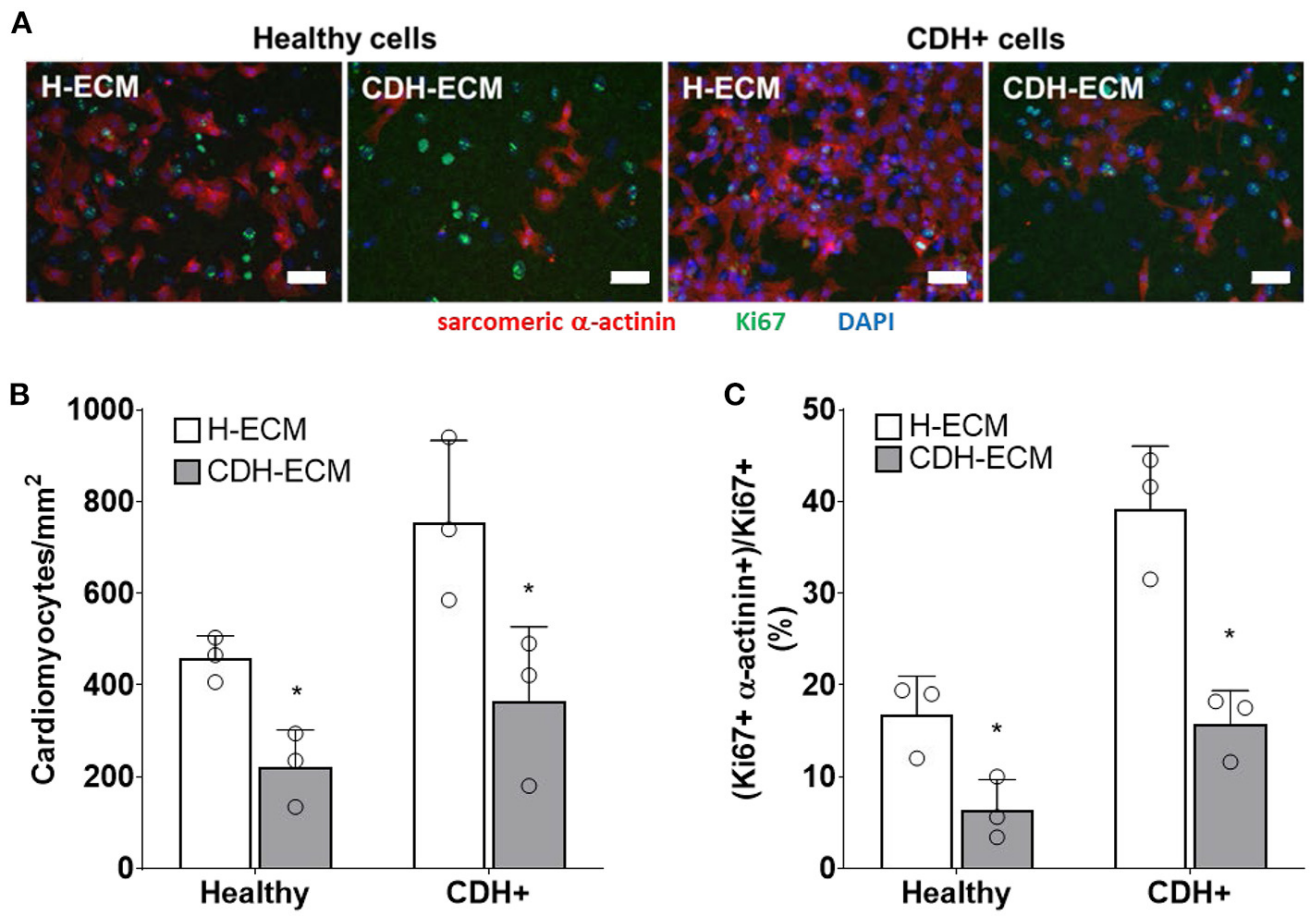
Cardiomyocyte response to ECM. **(A)** Representative images of cells on ECM derived from healthy hearts (H-ECM) and CDH+ hearts (CDH-ECM), stained for Hoechst (blue), Ki67 (green), and cardiac α-actinin (red). **(B)** Cardiomyocyte density and **(C)** cardiomyocyte proliferation were significantly decreased in both healthy and CDH+ populations on CDH-ECM after 4 days in culture. Graphs show mean ± SD (*n* = 3). In all cases * denotes *p* < 0.05.

Given that ECM composition was altered in CDH+ hearts (Figures 1E,F), we hypothesized that the ECM plays a role in the decreased proliferation observed in CDH-associated heart hypoplasia. Healthy and CDH+ cardiomyocytes were seeded onto healthy and CDH+ heart-derived ECM and cultured with serum-free medium. After 4 days, cardiomyocyte numbers were significantly higher on healthy ECM compared to CDH+ ECM for both healthy and CDH+ cardiomyocytes (Figures 5A,B). CDH+ cardiomyocytes were generally more proliferative than their healthy counterparts on either ECM. However, culture on CDH+ ECM resulted in significantly decreased cardiomyocyte proliferation for both healthy and CDH+ populations (Figure 5C). This data suggests that changes in ECM composition present in CDH+ hearts inhibits cardiomyocyte proliferation in CDH-associated heart hypoplasia.

### Cyclic mechanical loading promotes maturation of CDH+ cardiomyocytes

**Figure 6:**
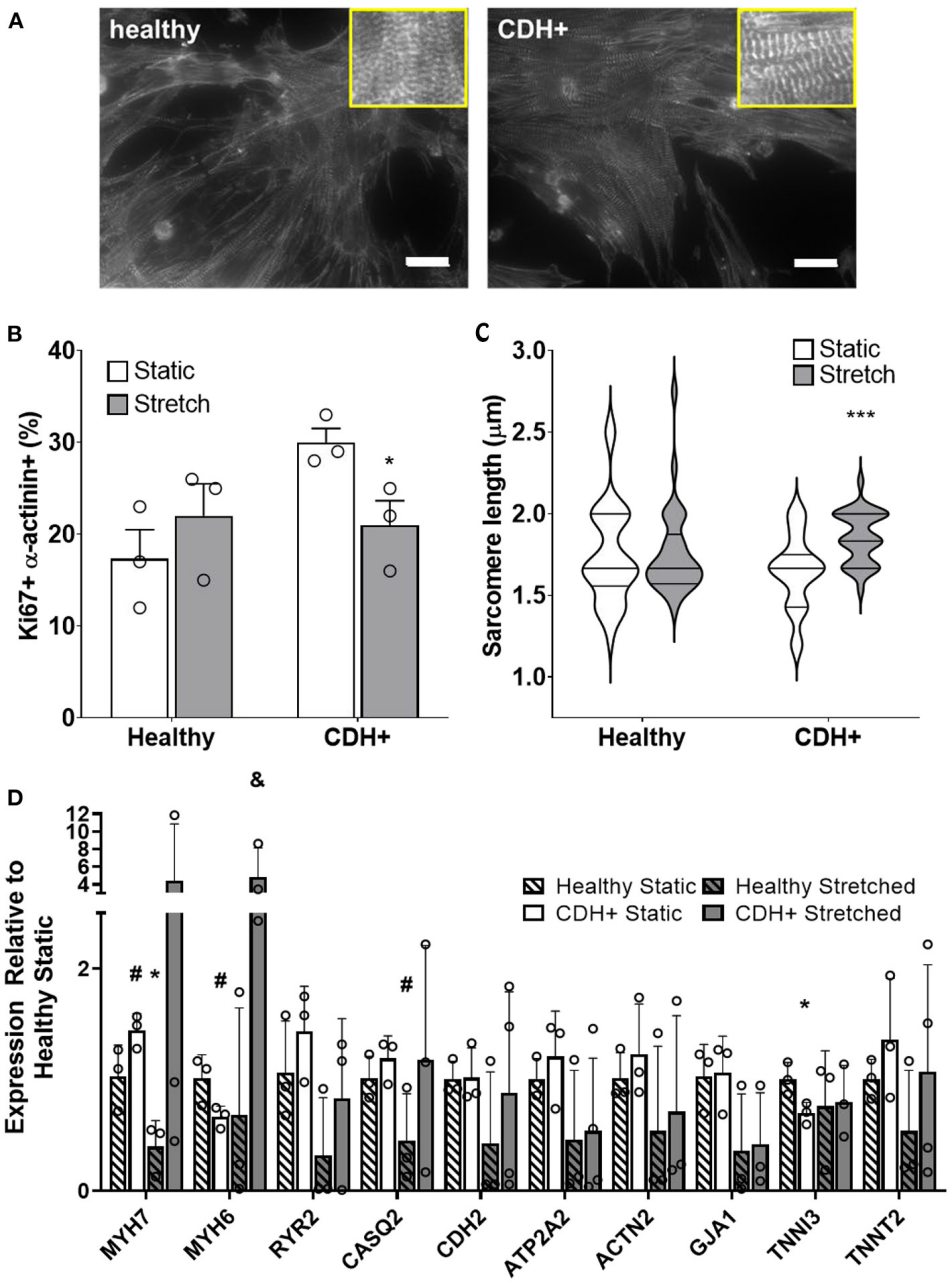
Cardiomyocyte responses to stretch. **(A)** Representative images of stretched cardiomyocytes (α-actinin stain). Insets show sarcomeres. Scale bars = 25 μm. **(B)** Cardiomyocyte proliferation was not significantly affected by stretch in healthy cells but was significantly decreased in CDH+ cells with stretch. Graph shows mean ± SD (*n* = 3). * Denotes *p* < 0.05. **(C)** CDH+ cardiomyocytes underwent lengthening of sarcomeres in response to stretch. Graph shows mean ± SEM (*n* ≥ 23). *** Denotes *p* < 0.001. **(D)** Panel of genes measured in healthy and CDH+ cells during both static conditions and following stretch. * Denotes *p* < 0.05 w.r.t Healthy Static, # denotes *p* < 0.1 w.r.t Healthy Static, and & denotes *p* < 0.1 w.r.t CDH+ Static.

Compression of the heart likely impedes the ability of the heart to undergo normal stretch in CDH (20). We hypothesized that mimicking physiological stretch *ex vivo* could promote CDH+ cardiomyocyte proliferation and maturation. For these studies, we used a frequency of 1 Hz at 5% amplitude. After 3 days of stretching (7 days in culture), we assessed sarcomere lengths (Figure 6A) and proliferation and we did see a significant decrease in cardiomyocyte specific proliferation in CDH+ cells (*p* < 0.05, Figure 6B). In addition, CDH+ cardiomyocytes had significantly longer sarcomeres after stretching compared to static controls (*p* < 0.001), while sarcomere lengths in healthy cells were unaffected by stretch (Figure 6C). Gene expression analysis of a panel of genes (Figure 6D), showed that CDH+ cells cultured under static conditions had lower expression of MHC6 and MHC7 than Healthy cells cultured statically (*p* < 0.1 for both). In addition, while MHC6 was lower in statically cultured cells, the addition of stretch led to a nearly significant increase in MHC6 in CDH+ cells (*p*<*0.1*), which also indicated increased maturity in CDH+ cells subjected to stretch. *p*-values for all comparisons of the gene expression data are shown in Table 2. Taken together, these results show that cyclic mechanical stretch promoted sarcomere maturation in CDH+ cardiomyocytes.

**Table 2:** Gene expression with stretch.

	**Static to static**	**Healthy static to stretch**	**CDH+ static to stretch**	**Stretch to stretch**
MYH7	0.09	0.04	0.47	0.34
MYH6	0.06	0.59	0.10	0.11
RYR2	0.36	0.14	0.27	0.37
CASQ2	0.33	0.10	0.99	0.31
CDH2	0.95	0.21	0.82	0.50
ATP2A2	0.48	0.22	0.18	0.88
Actn2	0.50	0.36	0.41	0.81
GJA1	0.88	0.12	0.12	0.89
TNNI3	0.04	0.46	0.63	0.92
TNNT2	0.36	0.23	0.67	0.44

p-values for t-tests between various groups for all genes measured in Figure 6D. Green denotes p < 0.05 while yellow denotes p ≤ 0.10.

## Discussion

The nitrofen model of CDH in rats is a valuable tool for studying molecular and cellular alterations in heart hypoplasia which are difficult in humans. Although a few groups have looked at changes in growth factors (20, 35, 36), and cardiac and ECM genes (20, 37, 38) in the nitrofen model of CDH, CDH+ cardiac cells have not been cultured or subjected to specific engineered environments to study their response to biochemical or biophysical cues. The key novel findings of this study are: (1) cell proliferation and cardiomyocyte maturity were decreased in CDH+ hearts compared to healthy; (2) in culture, CDH+ cardiomyocytes remained immature with increased proliferative potential compared to healthy cardiomyocytes; (3) ECM derived from CDH+ hearts significantly reduced both healthy and CDH+ cardiomyocyte proliferation compared healthy cardiac ECM; and (4) cyclic mechanical stretch promoted sarcomere maturation in CDH+ cardiomyocytes.

Given that the healthy heart undergoes a transition from hyperplastic to hypertrophic growth soon after birth (39, 40), cardiomyocyte proliferation and maturation are usually considered to be inversely correlated. However, we found that CDH+ hearts had smaller sarcomeres and reduced *mhc6* expression, suggesting a less mature state, while also having decreased proliferation compared to healthy controls- importantly these effects were not caused by a direct effect of nitrofen on cardiomyocytes in the developing hearts. Numerous external factors can influence cell behavior, such as growth factor signaling, the ECM, tissue stiffness, and the dynamic mechanical environment (6–12). While some of these cues are known to be altered in heart hypoplasia, it is unclear how their complex interactions could lead to concurrent decreased proliferation and maturity in the CDH+ heart. It was initially surprising to find that in culture the proliferation of CDH+ cardiomyocytes significantly exceeded that of healthy cardiomyocytes. It appears that the immature state of CDH+ cardiomyocytes is advantageous for recovered growth, as the removal of the hypoplastic environment allowed the cells to undergo a proliferative “burst” that would not be achievable by more mature myocytes. A similar mechanism may exist in young patients who undergo CDH repair, as recovery of heart dimensions have been observed in patients with mild to moderate heart hypoplasia (3). Given recent studies of young human hearts (8, 41), therapeutic strategies which promote cardiomyocyte proliferation will likely be most effective during early life.

Cardiac ECM influences cardiomyocyte proliferation (6, 7) but has not been well studied in the context of heart hypoplasia disorders. We found that culture on CDH+ ECM led to reduced proliferation of both healthy and CDH+ cardiomyocytes. This was intriguing since there were significant changes in only a small fraction of ECM components (Collagens IV, VI, and XIV, which were each < 3% of the ECM) relative to total composition. However, these proteins have important roles that could affect how cells sense and respond to the ECM (42–46). In CDH-associated heart hypoplasia and HLHS, two studies suggest that the ECM in hypoplastic hearts is less mature compared to healthy hearts (20, 21, 46). Specifically, Guarino et al. found reduced procollagen and tropoelastin in CDH+ fetal rat hearts and Davies et al. observed increased fibronectin and decreased collagen in HLHS hearts compared to non-HLHS patients. Tao et al. found that Col14a null mice displayed defects in ventricular morphogenesis and had significantly increased cardiomyocyte proliferation postnatally but a similar trend as in our CDH model toward decreased cardiomyocyte proliferation at E11.5 (45). Similarly, a recent study by Bousalis et al. observed increased expression of fibronectin, collagen IV, and integrin β-1 within *Nkx2-5* mutant embryonic mouse hearts compared to wild type hearts (46). Interactions between fibronectin and integrins can lead to downstream signaling cascades that control cell spreading, migration, and proliferation (47, 48). Our findings are counter-intuitive, as immature ECM has generally been found to promote proliferation (6–8, 49). The role of the ECM is more complex; future studies should explore the roles of specific peptides or interactions among ECM components to elucidate the nuances of the ECM in heart hypoplasia pathology and cardiomyocyte proliferation.

Normal stretching of the heart wall is diminished in CDH due to compression by visceral organs (20). We found that CDH+ cardiomyocytes had decreased proliferation and lengthened sarcomeres when stretched using a custom flexible membrane apparatus compared to static conditions; healthy cells, which were already relatively mature, did not further lengthen their sarcomeres under these cyclic loading conditions. Cyclic stretch has been used as a strategy to drive cardiomyocyte maturation in a number of tissue engineering applications (12, 50). Diminished mechanical movement experienced by cardiomyocytes within CDH+ hearts could be a potential mechanism of arrested cardiomyocyte maturity in CDH-associated heart hypoplasia and has also been implicated in hypoplastic left heart syndrome due to reduced blood flow in the left side of the heart (51). The role of mechanical forces is intriguing when contrasting CDH-associated heart hypoplasia and hypoplastic left heart syndrome. Although these two heart hypoplasia defects share some similar features such as reduced growth factors, altered ECM, and decreased cardiac transcription factors (5, 13, 20, 21, 52, 53), the outcomes after surgical repair are vastly different. Repair of CDH leads to removal of the compressive forces on the heart by intruding organs, and thus cardiac growth often normalizes (3). This is not the case for hypoplastic left heart syndrome: restoration of blood flow *via* fetal aortic valvuloplasty does not lead to recovered left ventricular growth (16). A deeper understanding of the specific underlying pathological mechanisms of these different forms of heart hypoplasia is needed.

Future direction related to this work could include a more detailed mechanistic study that attempts to dissect the specific ECM proteins or peptides that result in the reduction of proliferation in CDH+ hearts. For example, Yamashiro et al. found that stretch stimulation induces thrombospondin-1 secretion, which acts on cell adhesion plaques (54). While thrombospondin was not evident in our initial proteomics screen of heart ECM, it is possible that it is present in small amounts and it is also possible that it may be more expressed by the cells upon stretch in our system as well. Another potential avenue of future work related to this study would involve the study of samples taken from human hypoplastic heart patients. While these discarded samples that come from surgical repair often come from atrial tissue, it may still be a valuable source of tissue to assess changes in cardiomyocyte proliferation and ECM expression in the context of human congenital heart defects.

In conclusion, we have found that cardiomyocytes from CDH+ hearts are capable of robust proliferation, likely a result of their immature phenotype maintained during heart development. Our studies also point to altered ECM and mechanical forces as important environmental regulators of cardiomyocyte state in heart hypoplasia. Future work aimed at understanding these mechanisms could lead to novel biomechanical signaling-based therapies to improve cardiac growth and function in children born with hypoplastic hearts.

## Data availability statement

The original contributions presented in the study are included in the article/supplementary material, further inquiries can be directed to the corresponding author.

## Ethics statement

The animal study was reviewed and approved by Institutional Animal Care and Use Committee (IACUC) Tufts University.

## Author contributions

MW and CW designed and carried out the bulk of the experiments and wrote the manuscript. RW carried out the experiments and analysis related to cyclic stretch. LP assisted with cell isolations and experimental procedures for *in vitro* culture. KS and WS assisted with experimental design and analysis. LB assisted with experimental design, data interpretation and analysis, and writing/editing of the manuscript. All authors contributed to the article and approved the submitted version.

## Funding

This work was supported by an NIH/NHLBI postdoctoral fellowship (F32 HL112538) to CW, an American Heart Association Predoctoral Fellowship (14PRE19960001) to KS, NIH Institutional Research and Academic Career Development Awards (IRACDA) fellowship (K12GM074869), Training in Education and Critical Research Skills (TEACRS) to WS, and NIH/NHLBI (R21 HL115570), NSF (Award#: 1351241), DoD-CDMRP (Award#: W81XWH1610304), and American Heart Association (AHA, Award#: 20TPA35500082) to LB.

## Conflict of interest

The authors declare that the research was conducted in the absence of any commercial or financial relationships that could be construed as a potential conflict of interest.

## Publisher's note

All claims expressed in this article are solely those of the authors and do not necessarily represent those of their affiliated organizations, or those of the publisher, the editors and the reviewers. Any product that may be evaluated in this article, or claim that may be made by its manufacturer, is not guaranteed or endorsed by the publisher.
